# Longitudinal Effects of Academic Performance on Depression and Subjective Well-Being Among Students in China’s Elite Universities

**DOI:** 10.3390/bs16060863

**Published:** 2026-05-27

**Authors:** Xinqiao Liu, Xinyuan Zhang, Yunfeng Luo

**Affiliations:** 1 School of Education, Tianjin University, Tianjin 300350, China; 2 School of Public Administration, University of Electronic Science and Technology of China, Chengdu 611731, China; 3 Center for Israeli Studies, University of Electronic Science and Technology of China, Chengdu 611731, China

**Keywords:** academic performance, depression, subjective well-being, cross-lagged model, elite college

## Abstract

During the critical life transition of higher education, academic performance and mental health are two key factors that influence college students’ personal growth and future career development. Notably, there is ongoing debate regarding whether a bidirectional relationship exists between academic performance and mental health, and existing research lacks longitudinal evidence from students in China’s elite universities. As key indicators of mental health, the dynamic relationship between depression, subjective well-being, and academic performance warrants further investigation. This study utilizes data from two waves of the Beijing College Students Panel Survey, a large-scale longitudinal study of university students in Beijing that was launched in 2009. Using a sample of 874 students from five elite universities in Beijing, China, the study employed a cross-lagged model to examine the longitudinal bidirectional relationship between depression, subjective well-being and academic performance. The results show that academic performance is negatively correlated with depression and positively correlated with subjective well-being. Cross-lagged analysis further indicates that prior academic performance can predict subsequent depression (*β* = −0.066, *p* < 0.1) and subjective well-being (*β* = 0.082, *p* < 0.05), but there is insufficient evidence for the reverse predictive relationship (*p* > 0.1). These findings suggest that, for students in China’s elite universities, academic performance is a significant antecedent of subsequent mental health status. The conclusions emphasize the importance of enhancing academic support in elite universities to promote mental health and provide empirical evidence for constructing a collaborative support system that integrates academic and psychological aspects, as well as health communication strategies for behavioral change.

## 1. Introduction

Higher education is a crucial transitional stage in an individual’s life cycle and a key period that impacts their long-term quality of life and career development trajectory (Horowitz, 2018). Universities cultivate foundational competencies for future professionals to enter society and sustain their development by creating and providing academic environments (Rieckmann, 2012). Academic performance, as a core indicator for measuring the quality of college education and student academic achievement, has always been a focus of educational research and practice. Existing studies have indicated that academic performance is associated with various cognitive and non-cognitive factors (Rinn & Plucker, 2019), including relatively stable innate factors such as personality traits (Komarraju et al., 2009; Avram et al., 2019), perseverance (Wolters & Hussain, 2015; Whipple & Dimitrova-Grajzl, 2021), and socioeconomic status (Rheinschmidt & Mendoza-Denton, 2014; Y. Zhao et al., 2023; Gui et al., 2024), as well as psychological and behavioral variables characterized by dynamic changes, such as mental health status (J. Li et al., 2022; L. Zhao, 2023) and sleep quality (Okano et al., 2019; Gaultney, 2010). Simultaneously, academic performance has been confirmed to be a significant predictor of future career development for university students (T. Li & Zhang, 2010). Therefore, in-depth exploration of the influencing factors and subsequent effects of academic performance holds great significance for promoting students’ comprehensive development and enhancing the quality of higher education, with implications for health communication strategies aimed at behavioral change.

It is worth noting that mental health is a crucial component of college students’ development and plays a significant role in their academic performance. However, college students are considered a high-risk group for mental health issues. In recent years, the prevalence and complexity of their mental health problems have shown a concerning upward trend (Campbell et al., 2022; Ehl et al., 2025; X. Q. Liu et al., 2023, 2024; W. Z. Li et al., 2022). A nationwide survey of U.S. college students revealed that during the study period, mental health levels declined across all racial/ethnic groups, specifically manifested as decreased well-being and increased prevalence of depression, anxiety, eating disorders, and suicidal thoughts (Lipson et al., 2022). China, with the world’s largest higher education system, faces a similarly unignorable mental health crisis among college students (C. Wang et al., 2022; Hang et al., 2026; Chen et al., 2023). Meta-analyses have indicated that the prevalence of depression among Chinese college students is 28.4% (L. Gao et al., 2020), and the prevalence of anxiety is 25% (X. Wang & Liu, 2022). Additional research has found that female students and those from lower socioeconomic backgrounds are more susceptible to specific mental health challenges (Sun et al., 2022; W. Gao et al., 2020). Compared with students with good mental health, those who experience depression or reduced well-being have higher average absenteeism rates, lower learning efficiency, and are more likely to experience delays in normal academic progress (Wagner et al., 2022; Abu Ruz et al., 2018). Conversely, students facing academic difficulties and failures report higher levels of negative emotions, which may increase the risk of psychological issues such as depression (Erhun et al., 2022; Ajjawi et al., 2020).

Elite universities typically refer to higher education institutions that excel in academic achievement and rank among the top in world-class education rankings (X. Liu et al., 2024). With their outstanding educational standards, top-tier resources, and significant advantages in labor market returns (H. Li et al., 2012), elite universities naturally become aspirational goals and ideals for most students. However, students who successfully gain admission to elite universities face even more prominent academic competition and psychological pressure under the combined effects of rigorous academic standards, peer competition, and societal expectations from families (Bustamante et al., 2022). The excessive pursuit of excellence has become one of the key factors contributing to elevated mental health risks among students in high-achieving schools (Luthar et al., 2020). Additionally, the widespread emphasis on perfectionism and personal agency within elite academic cultures leads to stronger stigmatization of psychological issues in this group (Billings, 2021), which exacerbates their suboptimal mental health. To prevent more elite university students from falling into a vicious cycle of poor academic performance and psychological problems, it is necessary to clarify the mechanisms linking these two aspects.

Academic performance and well-being of university students have become key topics of ongoing concern for educators worldwide. Our study aims to provide new evidence and perspectives for exploring the relationship between these two factors. Therefore, a structural equation model is constructed to investigate in depth how academic performance is dynamically interrelated with depression and subjective well-being among elite university students within the Chinese cultural context. The findings of this study will provide a theoretical framework to guide the practical work of educators and policymakers.

## 2. Literature Review

### 2.1. Academic Performance and Depression

Depression is a negative emotional reaction and one of the most common mental health issues (Ramón-Arbués et al., 2020). Academic-related stress constitutes the primary non-pathological source of depression among college students (Deng et al., 2022; Jayanthi et al., 2015), although factors such as family expectations and unhealthy lifestyles also increase the risk of developing depression (X. Q. Liu et al., 2022; Foroughi et al., 2022). Research has indicated that depressive symptoms impair both cognitive and non-cognitive abilities, with an effect directly reflected in students’ academic performance (Eisenberg et al., 2009). Findings from multiple studies support this view, noting that high levels of depression are significantly associated with poor academic performance (Owens et al., 2012; Ma et al., 2018; de Sousa et al., 2018; Barbosa-Camacho et al., 2022). This association is more pronounced among students with poor academic performance (Y. Wang et al., 2023).

On the basis of these correlations, depression has been identified as a significant risk factor for poor academic performance (Mihailescu et al., 2016; Awadalla et al., 2020; Cao & Liu, 2025). The pathways of influence between the two still involve complex mediating mechanisms. Kayani et al. (2018) pointed out that physical exercise negatively impacts depression, indirectly promoting improvements in students’ academic performance. A longitudinal study further indicated that higher levels of depression can trigger stronger procrastination tendencies, leading to delayed task initiation, reduced execution efficiency, and ultimately causing persistent negative effects on academic achievement (Freyhofer et al., 2021). A study targeting medical students revealed that depression, anxiety, and stress had no direct significant effect on GPA, but the indirect impact through academic engagement was statistically significant (Sinval et al., 2025). The reverse predictive role of academic performance on depression has also garnered academic attention. A longitudinal study of Swedish adolescents revealed that lower final grades during compulsory education were associated with a higher risk of being diagnosed with depression in adulthood (Wallin et al., 2019). Huang conducted a meta-analysis on academic performance and later depression and reported an average effect size of *β* = 0.06 after controlling for prior academic performance (Huang, 2015). Evidence from China further highlighted that negative life events, including academic failure, can predict the exacerbation of depressive symptoms among college students, while worsening depression further diminishes academic engagement, creating a vicious cycle (Ji et al., 2021). On the basis of existing research findings, we propose the following hypotheses:

**Hypothesis** **1.** *There is a negative correlation between academic performance and depression among elite university students.*

**Hypothesis** **2.** *The academic performance of elite university students at T1 can negatively predict depression at T2.*

**Hypothesis** **3.** *The depression of elite university students at T1 can negatively predict academic performance at T2.*

In the above hypotheses, Hypotheses 1 and 3 serve to validate the direction of associations previously identified in existing research. Hypothesis 2, however, focuses on the reverse predictive effect of academic performance on subsequent depression, which constitutes the core focus of this study.

### 2.2. Academic Performance and Subjective Well-Being

In addition to traditional mental health symptoms, subjective well-being (SWB) has also been incorporated into research on its relationship with academic performance. SWB refers to an individual’s emotional experiences and cognitive evaluations of his or her life and typically encompasses three aspects: life satisfaction, positive affect, and negative affect (Diener et al., 2018a, 1999). High levels of SWB are often closely related to life characteristics associated with well-being judgments and can also predict positive life outcomes, such as health, income, and social relationships (Diener et al., 2018a, 2018b). In the university context, academic performance is a crucial indicator that influences self-identity and social evaluation among students and thus may be a significant factor associated with their SWB.

Existing research offers three explanations for the relationship between academic performance and SWB. First, SWB has a significant positive effect on academic performance. Evidence from Chinese middle school and university students indicates that higher SWB tends to predict better academic performance (X. K. Wang, 2025; Zhang & Chen, 2025). Second, academic performance can positively predict SWB. A German study revealed that students’ academic performance predicts changes in the cognitive variable (life satisfaction) within SWB (Crede et al., 2015). The bidirectional relationship between academic performance and SWB has also been explored, but the results have been conflicting. One study supported the view that SWB can predict subsequent academic performance but not vice versa (Wu et al., 2020). Another study suggested that academic performance can predict students’ subsequent SWB, but no predictive effect of SWB on academic performance was found (Yang et al., 2019; Steinmayr et al., 2016). Third, both academic performance and SWB are influenced by other variables (Steinmayr et al., 2018; Wong et al., 2024). Therefore, it is necessary to provide more empirical evidence for the study of the relationship between academic performance and SWB. We propose the following hypotheses:

**Hypothesis** **4.** *There is a positive correlation between academic performance and subjective well-being among elite university students.*

**Hypothesis** **5.** *The academic performance of elite university students at T1 can positively predict subjective well-being at T2.*

**Hypothesis** **6.** *The subjective well-being of elite university students at T1 can positively predict academic performance at T2*.

In the above hypotheses, Hypotheses 4 and 6 aim to verify the directions of influence previously reported in existing research. Hypothesis 5, meanwhile, focuses on the reverse predictive effect of academic performance on subsequent subjective well-being, representing another core area of exploration in this study.

### 2.3. The Present Study

Previous research has indicated that there may be a close and complex relationship between academic performance and psychological indicators such as depression and SWB, but the direction of influence and mechanisms have not yet reached a consensus. Furthermore, studies lack empirical evidence from student populations in elite universities within the Chinese cultural context. Under the influence of traditional Confucian culture and the intense competition in contemporary society, this group may exhibit unique psychological and behavioral characteristics. In summary, this study employs a cross-lagged model to examine the bidirectional relationships between depression, SWB and academic performance, delving into the intrinsic mechanisms of these variables within China’s specific cultural and environmental context. The research findings will hold significant reference value for promoting the comprehensive and healthy development of students in elite universities.

## 3. Materials and Methods

### 3.1. Procedure and Participants

The data for this study come from the Beijing College Students Panel Survey, a large-scale longitudinal study designed to comprehensively understand the academic and daily life circumstances of college students during their university years. In this research, we utilized two waves of survey data for modeling analysis, which were conducted during the junior and senior years. Using Project 985 universities as the exclusion criterion, we ultimately selected students from five elite universities, including Tsinghua University, Peking University, Renmin University of China, Beihang University, and Beijing Institute of Technology. Data were collected through self-reported questionnaires, and all participants voluntarily joined the study and provided informed consent.

In the first wave of the survey, 874 valid questionnaires were collected. In the sample, 60.53% were male, 92.45% were of Han nationality, 67.51% had no siblings, and 75.17% came from urban households. The age of the participants ranged from 18 to 27 years (M = 21.38, SD = 0.89). Detailed demographic characteristics are provided in Appendix A. The second wave, conducted one year later, successfully tracked 793 respondents, achieving a follow-up rate of 90.73%. T-tests comparing attrition and retained samples on key variables (gender, age, academic performance, depression, and subjective well-being) confirmed that the attrition met the assumption of missing data at random.

### 3.2. Measures

#### 3.2.1. Academic Performance

Academic performance is measured by the student’s ranking within the class. Specifically, we first calculate each student’s percentile rank on the basis of their original class rank and the total number of students in the class using the formula: 100* original class rank/total number of students in the class. The percentile rank is then divided into 10 levels, corresponding to intervals from the top 10% to the bottom 10%, and assigned values from 10 to 1. This means that a higher numerical value indicates better academic performance.

#### 3.2.2. Depression

Depression is measured using a subscale of the Depression Anxiety Stress Scale (DASS-42). This subscale contains 14 items (Crawford & Henry, 2003), and previous studies using this scale have reported good reliability and cross-cultural validity (X. Q. Liu & Wang, 2024). Each item is assessed using a 4-point Likert scale, where 0 represents “not applicable at all,” 1 represents “occasionally applicable or somewhat applicable,” 2 represents “very applicable or frequently applicable,” and 3 represents “extremely applicable or most applicable”. Students are asked to select the description that best matches their actual situation over the past week. The sum of the scores for all the items represents the total depression score, with higher scores indicating more severe depression. In this study, the reliability coefficients (α) for depression were 0.897 at T1 and 0.911 at T2, indicating good reliability.

#### 3.2.3. Subjective Well-Being

The measurement of subjective well-being was conducted using a single-item self-assessment method. This measurement tool requires students to comprehensively consider their own living conditions and evaluate their overall level of happiness. They need to provide a self-rating on a continuous scale from 0 to 100, with higher scores indicating stronger subjective well-being. This method, characterized by its simplicity and efficiency, has been widely applied in large-scale social surveys and can effectively reflect individuals’ overall perception of happiness (Abdel-Khalek, 2006).

#### 3.2.4. Other Covariates

The covariates in this study included age, gender, race, character, and family social status. Among these, age was obtained through students’ self-reported birth years and treated as a continuous variable in the analysis. Gender is a categorical variable consisting of male and female. Race is a categorical variable composed of Han nationality and national minorities. Character is an ordinal variable ranging from 1 (introverted) to 9 (extroverted). Students select a number that best represents their personality tendency on the basis of their self-perceived level of introversion or extroversion. Family social status reflects students’ subjective perceptions of their family’s social class. The numbers 1 to 5 represent the lower class, lower-middle class, middle class, upper-middle class, and upper class, respectively. In subsequent analyses, covariates were incorporated into the model to test their robustness.

### 3.3. Data Analysis

**Figure 1:**
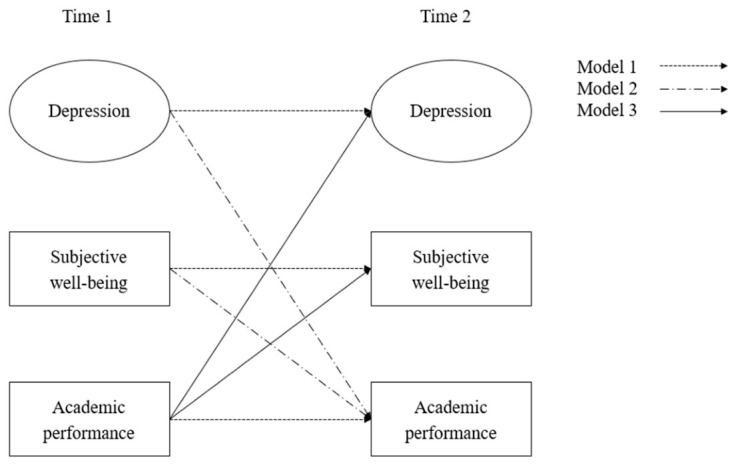
Cross-lagged models between depression and academic performance and between subjective well-being and academic performance.

This study used Stata 15.0 and Mplus 7.4 for data processing and analysis. First, we conducted a descriptive analysis of the main variables in the study and reported the correlations between academic performance, depression, and subjective well-being at T1 and T2. Subsequently, we constructed five models to systematically examine the complex relationships between the core variables, as shown in Figure 1.

Model 1 (M1) is an autoregressive model that includes regression paths from depression, SWB, and academic performance at T1 to their corresponding variables at T2. This model is used to examine the stability of the three core variables. Model 2 (M2) adds, in addition to the autoregressive paths, influence paths from depression and SWB at T1 to academic performance at T2, to investigate whether the former two variables have prospective predictive effects on the latter. Model 3 (M3) adds, in addition to the autoregressive paths, influence paths from academic performance at T1 to depression and SWB at T2, to test whether prior academic performance predicts later psychological indicators. Model 4 (M4) is a comprehensive model that includes both the autoregressive paths from M1 and the cross-lagged paths from M2 and M3. It is used to examine the bidirectional influence effects between the core variables. Model 5 (M5) further introduces covariates on the basis of M4 to assess the robustness of the model.

To assess the model’s fit, we reported various fit indices, including the chi-square value (χ^2^), root mean square error of approximation (RMSEA), standardized root mean square residual (SRMR), comparative fit index (CFI), and Tucker–Lewis index (TLI). In accordance with the recommendations of Hu and Bentler, a model fit is considered acceptable when RMSEA and SRMR are less than 0.10 and CFI and TLI are greater than 0.9. If RMSEA ≤ 0.06, SRMR ≤ 0.08, CFI ≥ 0.95, and TLI ≥ 0.95, the model fit is considered excellent (Hu & Bentler, 1998). In this study, the models are evaluated and compared on the basis of the aforementioned criteria.

## 4. Results

### 4.1. Descriptive Statistics and Correlation Analysis

The results of descriptive statistics and correlation analysis are shown in Table 1. At T1, the mean value of depression was 7.280 (SD = 6.711), the mean value of SWB was 84.161 (SD = 9.415), and the mean value of academic performance was 6.299 (SD = 2.467). By T2, the mean values of each variable were 7.120 (SD = 6.900) for depression, 84.673 (SD = 8.717) for SWB, and 6.402 (SD = 2.471) for academic performance. The sample exhibited a decrease in depression, along with increases in SWB and academic performance. The correlation results at the same time points showed that depression was significantly negatively correlated with SWB (r = −0.411 at T1, r = −0.450 at T2) and academic performance (r = −0.120 at T1, r = −0.121 at T2), while SWB was significantly positively correlated with academic performance (r = 0.185 at T1, r = 0.189 at T2). Consistent correlation patterns were also observed across time points. Our findings confirm Hypotheses 1 and 4. In other words, the academic performance of elite university students is negatively correlated with depression and positively correlated with SWB.

### 4.2. Model Comparisons

Table 2 reports the fit indices of the five models. Overall, all the models had RMSEA below 0.07, SRMR below 0.05, and CFI and TLI above 0.95, indicating relatively good fit levels. Specifically, M1 served as the baseline model and showed a relatively good fit (χ^2^ = 141.443, RMSEA = 0.064, SRMR = 0.045, CFI = 0.979, TLI = 0.968). Compared with M1, the chi-square difference in M2 was not significant (∆χ^2^ = 0.744, *p* > 0.05), indicating that the model was not improved. In contrast, both M3 (∆χ^2^ = 11.492, *p* < 0.05) and M4 (∆χ^2^ = 12.169, *p* < 0.05) showed significant differences compared to M1, indicating an improvement in the fit of both models. M5 demonstrated the best goodness-of-fit among all the models (χ^2^ = 144.380, RMSEA = 0.049, SRMR = 0.020, CFI = 0.981, TLI = 0.961), suggesting that the model results remained robust after controlling for demographic variables.

### 4.3. Cross-Lagged Analysis

Table 3 presents the autoregressive path coefficients and cross-lagged path coefficients of the five models. The results show that the autoregressive paths are significant in all the models (*p* < 0.01), indicating that the three core variables exhibit self-stability. The cross-lagged paths reveal unidirectional predictive relationships between the variables. In M3 and M4, academic performance at T1 significantly negatively predicts depression at T2 (*β* = −0.070, *p* < 0.05) and significantly positively predicts SWB at T2 (*β* = 0.104, *p* < 0.01), with the coefficients being identical in both models. However, the reverse predictive relationships are not supported. In M2 and M4, the predictive effect of depression at T1 on academic performance at T2 is not significant (*p* > 0.1), and the predictive effect of SWB at T1 on academic performance at T2 is also not significant (*p* > 0.1).

**Figure 2:**
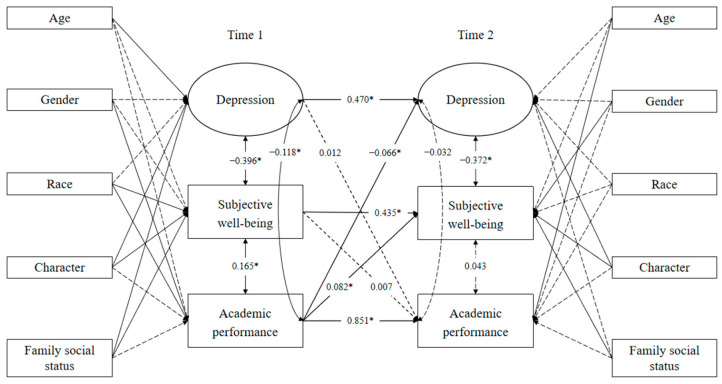
The model of depression, subjective well-being and academic performance with standardized coefficients. * 10% significance level.

This relationship pattern was ultimately confirmed in Model M5 after the covariates were incorporated, as shown in Figure 2. The solid lines indicate significant paths, while the dashed lines indicate non-significant paths. In M5, the autoregressive paths of the three variables were significant, with regression coefficients of 0.470 for depression (*p* < 0.01), 0.435 for SWB (*p* < 0.01), and 0.851 for academic performance (*p* < 0.01). Academic performance at T1 had a significant effect on depression (*β* = −0.066, *p* < 0.1) and SWB (*β* = 0.082, *p* < 0.05) at T2, confirming Hypotheses 2 and 5. However, depression and SWB at T1 did not significantly predict academic performance at T2 (*p* > 0.1), and Hypotheses 3 and 6 were not supported.

## 5. Discussion

Clarifying the complex relationships between academic performance and depression and between academic performance and SWB is crucial for understanding the psychological well-being and multifaceted challenges of university students. Although this topic has garnered academic attention, it remains inconclusive whether academic performance is a precursor or a consequence of psychological indicators such as depression and SWB, or whether a bidirectional mechanism exists. Therefore, this study employs a cross-lagged model to provide new evidence from a sample of elite university students in China for the ongoing debate over the directional relationship between academic performance and mental health.

Descriptive statistical results indicate that compared with their junior year, elite university students experience a reduction in depression levels and improvements in SWB and academic performance during their senior year. In China’s predominantly four-year higher education system, the junior year is a critical and challenging phase. Students must manage heavy academic workloads while preparing for career development choices such as post-graduate admissions and job hunting. Elite university students often hold themselves to higher standards and face greater pressure. Upon entering their senior year, as career paths become clearer, academic pressures and uncertainties about the future gradually diminish, which may explain the alleviation of depressive symptoms and improvements in SWB.

In the correlation analysis, academic performance showed a significant negative correlation with depression. Our finding is consistent with previous research (de Sousa et al., 2018; Owens et al., 2012; Ma et al., 2018), further validating the notion that poor academic performance is associated with high levels of depression. Simultaneously, we found a significant positive correlation between academic performance and SWB. Previously, Bücker et al. conducted a meta-analysis on the relationship between SWB and academic performance and reported an average association strength of 0.164, with a 95% confidence interval of [0.113, 0.216] (Bücker et al., 2018). The correlation coefficient observed in this study falls within this range, both contemporaneously and across periods. This finding not only confirms the small-to-moderate positive correlation between academic performance and SWB but also provides supplementary support from the student population of elite universities regarding their association.

This study utilized a cross-lagged model for analysis and revealed that the prior academic performance of elite university students could significantly predict depression and SWB one year later, but the reverse path was not supported. These findings align with conclusions from multiple studies (Wallin et al., 2019; Steinmayr et al., 2016; Yang et al., 2019; Huang, 2015). Research has indicated that academic performance is a primary source of stress for university students and a significant predictor of mental health status (Beiter et al., 2015). In the specific high-pressure environment of elite universities, academic performance is often deeply intertwined with students’ self-efficacy and social identity, so changes in this metric can trigger sustained psychological ripple effects. Notably, considering the grade background of the participants in this study, this impact holds unique explanatory significance. Academic performance during the junior year (T1) is directly linked to future graduate school recommendation qualifications, competitiveness in study abroad applications, and initial competitiveness in job hunting, rendering its effects persistent. These effects become even more pronounced when senior year (T2) outcomes for further education and employment are finalized. Therefore, it can be concluded that prior academic performance serves as a significant antecedent to later psychological states related to students’ personal development.

Although this study hypothesized that depression and SWB among elite university students would predict academic performance one year later, the data analysis results did not support this mechanism. On the one hand, this may be related to the research design, as the current research model may not fully capture the complex relationship between psychological indicators and academic performance. Students who successfully enter elite universities have passed through rigorous selection mechanisms and already possess strong psychological resilience and regulatory abilities (X. Liu et al., 2024). Even if depressive symptoms and changes in SWB occur, they may effectively mitigate the potential negative effects of psychological factors on academic performance through other pathways, such as resilience and self-efficacy (W. Q. Liu et al., 2024) and social support (Ji et al., 2021). On the other hand, compared to depression and SWB, other factors may play a more critical role in shaping and influencing academic performance (Steinmayr et al., 2016). For example, some studies suggest that motivation and attitude are key predictors of students’ academic performance (Ning & Downing, 2010). Therefore, the mechanisms through which psychological factors influence academic performance still require further exploration in future research.

## 6. Limitations

This study has five limitations that need to be addressed and improved in future research. First, the survey was conducted through self-reporting, which may have affected the accuracy of the results. Future studies could employ different survey methods to collect data. Second, the measurement instruments for all variables in this study were drawn from the existing Beijing College Students Panel Survey, and their selection was largely constrained by the existing design of this large-scale survey. Consideration should be given to adopting more sophisticated measurement tools, such as multidimensional scales and standardized scores, to further enhance the validity of the variables and their alignment with the theoretical framework. Third, the study included only two waves of data for longitudinal analysis, making it impossible to reveal the dynamic relationship between academic performance and depression/SWB over long-term development. Future research could adopt tracking designs with more time points to further explore the long-term interaction mechanisms among variables. Fourth, although the cross-lagged model helps explore the predictive relationships between variables, this study cannot establish causal inferences. Fifth, all the samples in this study were from five Project 985 universities in China. Whether the conclusions can be generalized to larger populations and more diverse cultural environments requires deeper exploration.

## 7. Implications for Educational Practice and Conclusions

### 7.1. Implications for Educational Practice

The study revealed that the academic performance of students in China’s elite universities is a significant antecedent variable for their subsequent mental health status. These findings highlight the profound connection between academic development and mental health in high-achieving groups, providing important insights for higher education practices.

First, create a supportive academic environment. Universities should fully recognize the profound impact of academic-related stress on students and engage in proactive and preventive measures. Educators should promptly identify students facing academic difficulties and provide them with academic resources and support, such as after-class tutoring, academic workshops, and peer-led courses. By enhancing students’ academic self-efficacy, the core sources of stress can be alleviated at the root, thereby preventing or reducing subsequent psychological issues.

Second, a collaborative support system that integrates academics and mental health should be established. Although the longitudinal impact of depression and SWB on academic performance is not significant, their strong correlation in cross-sectional analyses highlights their interconnectedness. Universities should break down the functional barriers between academic departments and psychological services to develop a collaborative mechanism. On the one hand, psychological support should be embedded in academic settings to provide regular mental health services. On the other hand, student development tracking surveys should be incorporated into the university’s routine operations, and periodic assessments of academic adaptability and mental health screenings should be conducted.

Third, create a new paradigm of intelligent mental health services. Big data and artificial intelligence provide unprecedented development opportunities for educational services. Universities must actively promote the intelligent transformation of mental health services. Under the premise of strictly adhering to data ethics and privacy protection, a dynamic student data monitoring and early warning system can be established. On the basis of the intelligent analysis results, personalized and precise resources and services can be provided to students with different needs. In the future, university mental health services should focus more on enhancing the accessibility and usability of AI-assisted mental health services, shifting from a passive response to proactive prevention, which reflects a positive psychology-informed approach to fostering well-being.

### 7.2. Conclusions

This study reached three conclusions. First, there is a negative correlation between academic performance and depression, and a positive correlation between academic performance and subjective well-being among students in China’s elite universities. Second, the academic performance of students in China’s elite universities can negatively predict depression one year later and positively predict subjective well-being one year later. Third, depression and subjective well-being among students in China’s elite universities do not have significant predictive effects on academic performance one year later.

These findings have practical implications. Mental health support systems in higher education institutions should not be limited to treatment alone but should extend to academic support, helping students receive academic assistance to prevent subsequent mental health issues.

## Figures and Tables

**Table 1:** Descriptive statistics and correlations for the main variables.

Variables	Mean	Standard Deviation	1	2	3	4	5	6
1. Depression-T1	7.280	6.711	1					
2. Subjective well-being-T1	84.161	9.415	−0.411 ***	1				
3. Academic performance-T1	6.299	2.467	−0.120 ***	0.185 ***	1			
4. Depression-T2	7.120	6.900	0.475 ***	−0.264 ***	−0.108 ***	1		
5. Subjective well-being-T2	84.673	8.717	−0.293 ***	0.492 ***	0.174 ***	−0.450 ***	1	
6. Academic performance-T2	6.402	2.471	−0.093 **	0.159 ***	0.856 ***	−0.121 ***	0.189 ***	1

Note: *** *p* < 0.01; ** *p* < 0.05.

**Table 2:** Fit indices of the models.

Model	χ^2^	df	RMSEA (90% CI)	SRMR	CFI	TLI	Comparison	∆χ^2^	*p*
M1	141.443	30	0.064 [0.054–0.075]	0.045	0.979	0.968			<0.05
M2	140.699	28	0.067 [0.056–0.078]	0.045	0.978	0.966	M1–M2	0.744	>0.05
M3	129.951	28	0.063 [0.053–0.075]	0.035	0.980	0.969	M1–M3	11.492	<0.05
M4	129.274	26	0.066 [0.055–0.078]	0.035	0.980	0.966	M1–M4	12.169	<0.05
M5	144.380	46	0.049 [0.040–0.059]	0.020	0.981	0.961	M1–M5	−2.937	<0.05

**Table 3:** Overview of the standardized stability and cross-lagged coefficients.

Model	Autoregressive Path	*β*	Cross-Lagged Path	*β*
M1	Dep(T1) → Dep(T2)	0.509 ***		
	SWB(T1) → SWB(T2)	0.492 ***		
	AP(T1) → AP(T2)	0.860 ***		
M2	Dep(T1) → Dep(T2)	0.509 ***	Dep(T1) → AP(T2)	0.014
	SWB(T1) → SWB(T2)	0.492 ***	SWB(T1) → AP(T2)	0.018
	AP(T1) → AP(T2)	0.859 ***		
M3	Dep(T1) → Dep(T2)	0.501 ***	AP(T1) → Dep(T2)	−0.070 **
	SWB(T1) → SWB(T2)	0.474 ***	AP(T1) → SWB(T2)	0.104 ***
	AP(T1) → AP(T2)	0.861 ***		
M4	Dep(T1) → Dep(T2)	0.501 ***	Dep(T1) → AP(T2)	0.014
	SWB(T1) → SWB(T2)	0.475 ***	SWB(T1) → AP(T2)	0.016
	AP(T1) → AP(T2)	0.860 ***	AP(T1) → Dep(T2)	−0.070 **
			AP(T1) → SWB(T2)	0.104 ***
M5	Dep(T1) → Dep(T2)	0.470 ***	Dep(T1) → AP(T2)	0.012
	SWB(T1) → SWB(T2)	0.435 ***	SWB(T1) → AP(T2)	0.007
	AP(T1) → AP(T2)	0.851 ***	AP(T1) → Dep(T2)	−0.066 *
			AP(T1) → SWB(T2)	0.082 **

Note: Dep denotes depression, SWB denotes subjective well-being, and AP denotes academic performance; *** *p* < 0.01; ** *p* < 0.05; * *p* < 0.1.

## Data Availability

The original contributions presented in this study are included in the article. Further inquiries can be directed to the corresponding author.

## Abbreviations

The following abbreviations are used in this manuscript:

SWB	Subjective well-being
DASS	Depression Anxiety Stress Scale
RMSEA	Root mean square error of approximation
SRMR	Standardized root mean square residual
CFI	Comparative fit index
TLI	Tucker–Lewis index

## Appendix A

**Table A1:** Characteristics of the participants.

Variable	Category	Number	Percent	M ± SD	Range
Gender	Female	345	39.47%		
	Male	529	60.53%		
Age		874		21.38 ± 0.89	18–27
Nationality	Han nationality	808	92.45%		
	National minority	66	7.55%		
Character		874		5.51 ± 1.63	1–9
Sibling	Yes	284	32.49%		
	No	590	67.51%		
Hometown location	Urban	657	75.17%		
	Rural	217	24.83%		
Father’s education level	Never accept formal education	4	0.51%	13.57 ± 3.62	0–19
	Primary school	37	4.67%		
	Middle school	133	16.79%		
	Senior high school	194	24.49%		
	Bachelor’s degree or associate degree	351	44.32%		
	Master’s degree and above	73	9.22%		
Mother’s education level	Never accept formal education	23	2.92%	12.43 ± 4.05	0–19
	Primary school	71	9.00%		
	Middle school	122	15.46%		
	Senior high school	238	30.16%		
	Bachelor’s degree or associate degree	313	39.67%		
	Master’s degree and above	22	2.79%		
Family economic status	Upper	4	0.46%		
	Upper-middle	156	17.85%		
	Middle	421	48.17%		
	Middle-low	238	27.23%		
	Low	55	6.29%		
Family social status	Upper	9	1.03%		
	Upper-middle	233	26.66%		
	Middle	410	46.91%		
	Middle-low	166	18.99%		
	Low	56	6.41%		

Note: The average parental education level refers to the mean of their years of education, while the range indicates the variation interval of education years. The assignment of years of education is as follows: no formal education is given a score of 0, primary school is given a score of 6, junior high school is given a score of 9, senior high school (including vocational high school and technical secondary school) is given a score of 12, university (including college) is given a score of 16, and postgraduate or above is given a score of 19.

## References

[B1-behavsci-16-00863] Abdel-Khalek A. M. (2006). Measuring happiness with a single-item scale. Social Behavior and Personality.

[B2-behavsci-16-00863] Abu Ruz M. E., Al-Akash H. Y., Jarrah S. (2018). Persistent (anxiety and depression) affected academic achievement and absenteeism in nursing students. The Open Nursing Journal.

[B3-behavsci-16-00863] Ajjawi R., Dracup M., Zacharias N., Bennett S., Boud D. (2020). Persisting students’ explanations of and emotional responses to academic failure. Higher Education Research & Development.

[B4-behavsci-16-00863] Avram E., Burtaverde V., Zanfirescu A. S. (2019). The incremental validity of career adaptability in predicting academic performance. Social Psychology of Education.

[B5-behavsci-16-00863] Awadalla S., Davies E. B., Glazebrook C. (2020). A longitudinal cohort study to explore the relationship between depression, anxiety and academic performance among Emirati university students. BMC Psychiatry.

[B6-behavsci-16-00863] Barbosa-Camacho F. J., Romero-Limón O. M., Ibarrola-Peña J. C., Almanza-Mena Y. L., Pintor-Belmontes K. J., Sánchez-López V. A., Chejfec-Ciociano J. M., Guzmán-Ramírez B. G., Sapién-Fernández J. H., Guzmán-Ruvalcaba M. J., Nájar-Hinojosa R., Ochoa-Rodriguez I., Cueto-Valadez T. A., Cueto-Valadez A. E., Fuentes-Orozco C., Cortés-Flores A. O., Miranda-Ackerman R. C., Cervantes-Cardona G. A., Cervantes-Guevara G., González-Ojeda A. (2022). Depression, anxiety, and academic performance in COVID-19: A cross-sectional study. BMC Psychiatry.

[B7-behavsci-16-00863] Beiter R., Nash R., McCrady M., Rhoades D., Linscomb M., Clarahan M., Sammut S. (2015). The prevalence and correlates of depression, anxiety, and stress in a sample of college students. Journal of Affective Disorders.

[B8-behavsci-16-00863] Billings K. R. (2021). Stigma in class: Mental illness, social status, and tokenism in elite college culture. Sociological Perspectives.

[B9-behavsci-16-00863] Bustamante C. M. V., Coombs G., Rahimi-Eichi H., Mair P., Onnela J. P., Baker J. T., Buckner R. L. (2022). Fluctuations in behavior and affect in college students measured using deep phenotyping. Scientific Reports.

[B10-behavsci-16-00863] Bücker S., Nuraydin S., Simonsmeier B. A., Schneider M., Luhmann M. (2018). Subjective well-being and academic achievement: A meta-analysis. Journal of Research in Personality.

[B11-behavsci-16-00863] Campbell F., Blank L., Cantrell A., Baxter S., Blackmore C., Dixon J., Goyder E. (2022). Factors that influence mental health of university and college students in the UK: A systematic review. BMC Public Health.

[B12-behavsci-16-00863] Cao X. J., Liu X. Q. (2025). Effect of depressive symptoms and learning difficulty on academic achievement among adolescents in China: A cross-lagged panel study. International Journal of Psychology.

[B13-behavsci-16-00863] Chen C. P., He Z. G., Xu B. N., Shao J. Y., Wang D. F. (2023). A latent profile analysis of sleep disturbance in relation to mental health among college students in China. Frontiers in Public Health.

[B14-behavsci-16-00863] Crawford J. R., Henry J. D. (2003). The depression anxiety stress scales (DASS): Normative data and latent structure in a large non-clinical sample. British Journal of Clinical Psychology.

[B15-behavsci-16-00863] Crede J., Wirthwein L., McElvany N., Steinmayr R. (2015). Adolescents’ academic achievement and life satisfaction: The role of parents’ education. Frontiers in Psychology.

[B16-behavsci-16-00863] Deng Y. W., Cherian J., Khan N. U. N., Kumari K., Sial M. S., Comite U., Gavurova B., Popp J. (2022). Family and academic stress and their impact on students’ depression level and academic performance. Frontiers in Psychiatry.

[B17-behavsci-16-00863] de Sousa J. M., Moreira C. A., Telles-Correia D. (2018). Anxiety, depression and academic performance: A study amongst portuguese medical students versus non-medical students. Acta Medica Portuguesa.

[B18-behavsci-16-00863] Diener E., Lucas R. E., Oishi S. (2018a). Advances and open questions in the science of subjective well-being. Collabra-Psychology.

[B19-behavsci-16-00863] Diener E., Oishi S., Tay L. (2018b). Advances in subjective well-being research. Nature Human Behaviour.

[B20-behavsci-16-00863] Diener E., Suh E. M., Lucas R. E., Smith H. L. (1999). Subjective well-being: Three decades of progress. Psychological Bulletin.

[B21-behavsci-16-00863] Ehl L., Scheiner C., Wasserscheid A., Hein G., Gamer M., Bürger A. (2025). German college students’ mental health state and their willingness to use mental health prevention: An online survey during the COVID-19 pandemic. Heliyon.

[B22-behavsci-16-00863] Eisenberg D., Golberstein E., Hunt J. B. (2009). Mental health and academic success in college. B E Journal of Economic Analysis & Policy.

[B23-behavsci-16-00863] Erhun W. O., Jegede A. O., Ojelabi J. A. (2022). Implications of failure on students who have repeated a class in a faculty of pharmacy. Currents in Pharmacy Teaching and Learning.

[B24-behavsci-16-00863] Foroughi B., Griffiths M. D., Iranmanesh M., Salamzadeh Y. (2022). Associations between instagram addiction, academic performance, social anxiety, depression, and life satisfaction among university students. International Journal of Mental Health and Addiction.

[B25-behavsci-16-00863] Freyhofer S., Ziegler N., de Jong E. M., Schippers M. C. (2021). Depression and anxiety in times of COVID-19: How coping strategies and loneliness relate to mental health outcomes and academic performance. Frontiers in Psychology.

[B26-behavsci-16-00863] Gao L., Xie Y. C., Jia C. H., Wang W. (2020). Prevalence of depression among Chinese university students: A systematic review and meta-analysis. Scientific Reports.

[B27-behavsci-16-00863] Gao W., Ping S., Liu X. (2020). Gender differences in depression, anxiety, and stress among college students: A longitudinal study from China. Journal of Affective Disorders.

[B28-behavsci-16-00863] Gaultney J. F. (2010). The prevalence of sleep disorders in college students: Impact on academic performance. Journal of American College Health.

[B29-behavsci-16-00863] Gui P., Alam G. M., Hassan A. B. (2024). Whether socioeconomic status matters in accessing residential college: Role of RC in addressing academic achievement gaps to ensure sustainable education. Sustainability.

[B30-behavsci-16-00863] Hang Y., Feng Y., Zou H., Zhao H., Liu F., Qin X., Qiao Z. (2026). Tracing two decades: Non-linear temporal trends in mental health among Chinese university students (2005–2024). Journal of Affective Disorders.

[B31-behavsci-16-00863] Horowitz J. (2018). Relative education and the advantage of a college degree. American Sociological Review.

[B32-behavsci-16-00863] Hu L.-T., Bentler P. M. (1998). Fit indices in covariance structure modeling: Sensitivity to underparameterized model misspecification. Psychological Methods.

[B33-behavsci-16-00863] Huang C. (2015). Academic achievement and subsequent depression: A meta-analysis of longitudinal studies. Journal of Child and Family Studies.

[B34-behavsci-16-00863] Jayanthi P., Thirunavukarasu M., Rajkumar R. (2015). Academic stress and depression among adolescents: A cross-sectional study. Indian Pediatrics.

[B35-behavsci-16-00863] Ji L., Chen C. F., Hou B. Y., Ren D. C., Yuan F., Liu L. J., Bi Y., Guo Z. M., Yang F. P., Wu X., Li X., Liu C., Zuo Z., Zhang R., Yi Z., Xu Y., He L., Shi Y., Yu T., He G. (2021). A study of negative life events driven depressive symptoms and academic engagement in Chinese college students. Scientific Reports.

[B36-behavsci-16-00863] Kayani S., Kiyani T., Wang J., Sánchez M. L. Z., Kayani S., Qurban H. (2018). Physical activity and academic performance: The mediating effect of self-esteem and depression. Sustainability.

[B37-behavsci-16-00863] Komarraju M., Karau S. J., Schmeck R. R. (2009). Role of the big five personality traits in predicting college students’ academic motivation and achievement. Learning and Individual Differences.

[B38-behavsci-16-00863] Li H., Meng L., Shi X., Wu B. (2012). Does attending elite colleges pay in China?. Journal of Comparative Economics.

[B39-behavsci-16-00863] Li J., Yang D., Hu Z. (2022). Wuhan college students’ self-directed learning and academic performance: Chain-mediating roles of optimism and mental health. Frontiers in Psychology.

[B40-behavsci-16-00863] Li T., Zhang J. (2010). What determines employment opportunity for college graduates in China after higher education reform?. China Economic Review.

[B41-behavsci-16-00863] Li W. Z., Zhao Z. Y., Chen D. J., Peng Y., Lu Z. X. (2022). Prevalence and associated factors of depression and anxiety symptoms among college students: A systematic review and meta-analysis. Journal of Child Psychology and Psychiatry.

[B42-behavsci-16-00863] Lipson S. K., Zhou S., Abelson S., Heinze J., Jirsa M., Morigney J., Patterson A., Singh M., Eisenberg D. (2022). Trends in college student mental health and help-seeking by race/ethnicity: Findings from the national healthy minds study, 2013–2021. Journal of Affective Disorders.

[B43-behavsci-16-00863] Liu W. Q., Zhang R. Y., Wang H., Rule A., Wang M., Abbey C., Singh M. K., Rozelle S., She X. S., Tong L. (2024). Association between anxiety, depression symptoms, and academic burnout among Chinese students: The mediating role of resilience and self-efficacy. BMC Psychology.

[B44-behavsci-16-00863] Liu X., Zhu C., Dong Z., Luo Y. (2024). The relationship between stress and academic self-efficacy among students at elite colleges: A longitudinal analysis. Behavioral Sciences.

[B45-behavsci-16-00863] Liu X. Q., Guo Y. X., Zhang W. J., Gao W. J. (2022). Influencing factors, prediction and prevention of depression in college students: A literature review. World Journal of Psychiatry.

[B46-behavsci-16-00863] Liu X. Q., Li Y., Gao W. J. (2024). Subjective well-being of college students: Developmental trajectories, predictors, and risk for depression. Journal of Psychology in Africa.

[B47-behavsci-16-00863] Liu X. Q., Wang J. X. (2024). Depression, anxiety, and student satisfaction with university life among college students: A cross-lagged study. Humanities & Social Sciences Communications.

[B48-behavsci-16-00863] Liu X. Q., Zhang Y. F., Gao W. J., Cao X. J. (2023). Developmental trajectories of depression, anxiety, and stress among college students: A piecewise growth mixture model analysis. Humanities & Social Sciences Communications.

[B49-behavsci-16-00863] Luthar S. S., Kumar N. L., Zillmer N. (2020). High-achieving schools connote risks for adolescents: Problems documented, processes implicated, and directions for interventions. American Psychologist.

[B50-behavsci-16-00863] Ma Y., Siu A., Tse W. S. (2018). The role of high parental expectations in adolescents’ academic performance and depression in Hong Kong. Journal of Family Issues.

[B51-behavsci-16-00863] Mihailescu A. I., Diaconescu L. V., Ciobanu A. M., Donisan T., Mihailescu C. (2016). The impact of anxiety and depression on academic performance in undergraduate medical students. European Psychiatry.

[B52-behavsci-16-00863] Ning H. K., Downing K. (2010). The reciprocal relationship between motivation and self-regulation: A longitudinal study on academic performance. Learning and Individual Differences.

[B53-behavsci-16-00863] Okano K., Kaczmarzyk J. R., Dave N., Gabrieli J. D. E., Grossman J. C. (2019). Sleep quality, duration, and consistency are associated with better academic performance in college students. Npj Science of Learning.

[B54-behavsci-16-00863] Owens M., Stevenson J., Hadwin J. A., Norgate R. (2012). Anxiety and depression in academic performance: An exploration of the mediating factors of worry and working memory. School Psychology International.

[B55-behavsci-16-00863] Ramón-Arbués E., Gea-Caballero V., Granada-López J. M., Juárez-Vela R., Pellicer-García B., Antón-Solanas I. (2020). The prevalence of depression, anxiety and stress and their associated factors in college students. International Journal of Environmental Research and Public Health.

[B56-behavsci-16-00863] Rheinschmidt M. L., Mendoza-Denton R. (2014). Social class and academic achievement in college: The interplay of rejection sensitivity and entity beliefs. Journal of Personality and Social Psychology.

[B57-behavsci-16-00863] Rieckmann M. (2012). Future-oriented higher education: Which key competencies should be fostered through university teaching and learning?. Futures.

[B58-behavsci-16-00863] Rinn A., Plucker J. (2019). High-ability college students and undergraduate honors programs: A systematic review. Journal for the Education of the Gifted.

[B59-behavsci-16-00863] Sinval J., Oliveira P., Novais F., Almeida C. M., Telles-Correia D. (2025). Exploring the impact of depression, anxiety, stress, academic engagement, and dropout intention on medical students’ academic performance: A prospective study. Journal of Affective Disorders.

[B60-behavsci-16-00863] Steinmayr R., Crede J., McElvany N., Wirthwein L. (2016). Subjective well-being, test anxiety, academic achievement: Testing for reciprocal effects. Frontiers in Psychology.

[B61-behavsci-16-00863] Steinmayr R., Heyder A., Naumburg C., Michels J., Wirthwein L. (2018). School-related and individual predictors of subjective well-being and academic achievement. Frontiers in Psychology.

[B62-behavsci-16-00863] Sun X., Wang Z.-J., Li Y.-Y., Chan K. Q., Miao X.-Y., Zhao S., Wu Y.-Q., Li Z., Wu B.-M. (2022). Trends of college students’ mental health from 2005 to 2019 and its rural–urban disparities in China. Journal of Affective Disorders.

[B63-behavsci-16-00863] Wagner F., Wagner R. G., Kolanisi U., Makuapane L., Masango M., Gómez-Olivé F. X. (2022). The relationship between depression symptoms and academic performance among first-year undergraduate students at a South African university: A cross-sectional study. BMC Public Health.

[B64-behavsci-16-00863] Wallin A. S., Koupil I., Gustafsson J. E., Zammit S., Allebeck P., Falkstedt D. (2019). Academic performance, externalizing disorders and depression: 26,000 adolescents followed into adulthood. Social Psychiatry and Psychiatric Epidemiology.

[B65-behavsci-16-00863] Wang C., Yan S., Jiang H., Guo Y., Gan Y., Lv C., Lu Z. (2022). Socio-demographic characteristics, lifestyles, social support quality and mental health in college students: A cross-sectional study. BMC Public Health.

[B66-behavsci-16-00863] Wang X., Liu Q. (2022). Prevalence of anxiety symptoms among Chinese university students amid the COVID-19 pandemic: A systematic review and meta-analysis. Heliyon.

[B67-behavsci-16-00863] Wang X. K. (2025). Exploring the impact of mindfulness, subjective well-being, and music engagement on academic performance of students in higher educational institutions. Humanities & Social Sciences Communications.

[B68-behavsci-16-00863] Wang Y., Zhang S., Liu X. G., Shi H. Y., Deng X. Y. (2023). Differences in central symptoms of anxiety and depression between college students with different academic performance: A network analysis. Frontiers in Psychology.

[B69-behavsci-16-00863] Whipple S. S., Dimitrova-Grajzl V. (2021). Grit, fit, gender, and academic achievement among first-year college students. Psychology in the Schools.

[B70-behavsci-16-00863] Wolters C., Hussain M. (2015). Investigating grit and its relations with college students’ self-regulated learning and academic achievement. Metacognition and Learning.

[B71-behavsci-16-00863] Wong Z. Y., Liem G. A. D., Chan M., Datu J. A. D. (2024). Student engagement and its association with academic achievement and subjective well-being: A systematic review and meta-analysis. Journal of Educational Psychology.

[B72-behavsci-16-00863] Wu X., Gai X., Wang W. (2020). Subjective well-being and academic performance among middle schoolers: A two-wave longitudinal study. Journal of Adolescence.

[B73-behavsci-16-00863] Yang Q., Tian L. L., Huebner E. S., Zhu X. X. (2019). Relations among academic achievement, self-esteem, and subjective well-being in school among elementary school students: A longitudinal mediation model. School Psychology.

[B74-behavsci-16-00863] Zhang T. Y., Chen X. (2025). The mediating role of learning self-efficacy in the relationship between subjective well-being and academic performance in children. Frontiers in Psychology.

[B75-behavsci-16-00863] Zhao L. (2023). Social media addiction and its impact on college students’ academic performance: The mediating role of stress. Asia-Pacific Education Researcher.

[B76-behavsci-16-00863] Zhao Y., Wang Z., Ren Z. Y. (2023). Research on the influence of family capital on academic achievement of first-generation college students in China. Frontiers in Psychology.

